# Development and validation of a model for predicting acute kidney injury after cardiac surgery in patients of advanced age

**DOI:** 10.1111/jocs.15249

**Published:** 2020-12-12

**Authors:** Penghua Hu, Yuanhan Chen, Yanhua Wu, Li Song, Li Zhang, Zhilian Li, Lei Fu, Shuangxin Liu, Zhiming Ye, Wei Shi, Xinling Liang

**Affiliations:** ^1^ The Second School of Clinical Medicine Southern Medical University Guangzhou China; ^2^ Division of Nephrology, Guangdong Provincial People's Hospital Guangdong Academy of Medical Sciences Guangzhou China

**Keywords:** acute kidney injury, advanced age, cardiac surgery, risk assessment

## Abstract

**Objective:**

To develop a clinical model for predicting postoperative acute kidney injury (AKI) in patients of advanced age undergoing cardiac surgery.

**Methods:**

A total of 848 patients (aged ≥ 60 years) undergoing cardiac surgery were consecutively enrolled. Among them, 597 were randomly selected for the development set and the remaining 251 for the validation set. AKI was the primary outcome. To develop a model for predicting AKI, visualized as a nomogram, we performed logistic regression with variables selected by Lasso regression analysis. The discrimination, calibration, and clinical usefulness of the new model were assessed and compared with those of Cleveland Clinic score and Simplified Renal Index (SRI) score in the validation set.

**Results:**

The incidence of AKI was 61.8% in the development set. The new model included seven variables including preoperative serum creatinine, hypertension, preoperative uric acid, New York Heart Association classification ≥ 3, cardiopulmonary bypass time > 120 min, intraoperative red blood cell transfusion, and postoperative prolonged mechanical ventilation. In the validation set, the areas under the receiver operating characteristic curves for assessing discrimination of the new model, Cleveland Clinic score, and SRI score were 0.801, 0.670, and 0.627, respectively. Compared with the other two scores, the new model presented excellent calibration according to the calibration curves. Decision curve analysis presented the new model was more clinically useful than the other two scores.

**Conclusions:**

We developed and validated a new model for predicting AKI after cardiac surgery in patients of advanced age, which may help clinicians assess patients' risk for AKI.

## 1 INTRODUCTION

With the continued expansion of the aging population and the higher prevalence of cardiovascular disorders in the advanced age population, the number of advanced age patients who need cardiac surgery has been rising in recent years.^1^, ^2^ Currently, cardiac surgeries offered to patients of advanced age include coronary artery bypass graft (CABG) surgery and valve surgery. Given the higher rate of postoperative complications,^3^ the quality of life of patients of advanced age after cardiac surgery is more likely to be compromised than that of younger patients.^4^ Acute kidney injury (AKI) is one of the major complications associated with cardiac surgery. Based on the type of cardiac surgery and the definition of AKI, the AKI incidence after cardiac surgery ranges from 20% to 70%.^5^, ^6^ AKI may compromise patient's quality of life and is linked to high mortality, all of which pose a heavy financial burden on society and families.^7^

An easily calculable clinical risk model based on the basic characteristics of patients and clinical data that are easily available during the perioperative period may facilitate clinical decision‐making, patient counseling, and medical optimization. To date, several models^8^, ^9^, ^10^, ^11^, ^12^ have been developed, such as Simplified Renal Index (SRI) score^9^ and Cleveland Clinic score,^11^ which have been frequently validated in European and American patients. However, most existing models were designed to predict AKI requiring renal replacement therapy. Compared with mild AKI, which does not require dialysis, the incidence of renal replacement therapy is low and occurs late in clinical practice, which limits the application of these models. Because a mild increase in serum creatinine is also related to a poor prognosis,^13^ it is clinically imperative to develop a model to predict all stages of AKI. In addition, recent studies have uncovered several new independent risk factors for AKI after cardiac surgery, including uric acid level^14^ and red blood cell (RBC) transfusion,^15^ which were not included in the development of the aforementioned predictive models. Furthermore, cardiac surgery is booming in developing countries due to the development of medical technology. The proportions of population race, comorbidities, and valve surgery are quite different between developing countries, such as China, and those of the existing model derivation cohorts.^16^ Lastly, patients of advanced age may have different comorbidities and risk factors for AKI than young patients.^17^ Therefore, there is a specific need for an appropriate model that can predict AKI in patients of advanced age undergoing cardiac surgery to guide their clinical management.

The aim of this study was to construct a model for predicting AKI after cardiac surgery in patients of advanced age using readily available clinical data and to compare this new model with two previous models, the SRI score and Cleveland Clinic score, with regard to their discrimination, calibration, and clinical usefulness for predicting AKI risk.

## 2 MATERIALS AND METHODS

### 2.1 Study population

**Figure 1:**
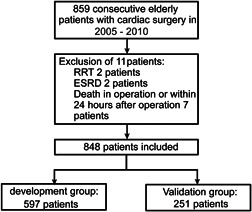
Flowchart outlining participant selection. ESRD, end‐stage renal disease; RRT, renal replacement therapy

Patients of advanced age (aged ≥ 60 years) who underwent valve surgery and/or CABG with cardiopulmonary bypass (CPB) at the Guangdong Provincial People's Hospital between January 2005 and December 2010 were consecutively enrolled. Patients with any one of the following conditions were excluded: preoperative renal replacement therapy, preoperative end‐stage renal disease (estimated glomerular filtration rate [eGFR] based on the Chronic Kidney Disease‐Epidemiology Collaboration formula,^18^ less than 15 ml/min × 1.73 m^2^), or death during or within 24 h after surgery. If some patients had more than one cardiac surgery procedures performed during the study period, only the first operation was included for analysis. Initially, 859 patients were registered. After application of the inclusion and exclusion criteria, 11 patients were excluded, and 848 were enrolled. The included study population was randomly assigned to the development set and validation set at a ratio of 7:3. Figure 1 presents a detailed flow chart of patient selection.

This study complied with the Declaration of Helsinki and was approved by the Ethics Committee of Guangdong Provincial People's Hospital without the need for signed informed consent from the participants (No. GDREC2018416H). Also, as this was a retrospective study, all subject identification information was removed before analysis.

### 2.2 Data collection

The data of all participants were collected retrospectively through electronic health records established in our hospital. The potential variables used to develop this new prediction model were selected based on the well‐recognized AKI risk factors in the field.^19^ Demographic characteristics included gender, age, weight, and height. Preoperative data included diabetes, peripheral vascular disease, cerebrovascular disease, hypertension, chronic obstructive pulmonary disease, recent myocardial infarction (occurred within 1 month before surgery), previous heart surgery, intra‐aortic balloon pump (IABP), recent contrast agent exposure (within 7 days before surgery), New York Heart Association (NYHA) functional classification, left ventricular ejection fraction (LVEF), baseline eGFR, anemia, emergency surgery, uric acid level, proteinuria (defined based on the urine protein results obtained from routine urine exam at the preoperative admission: mild proteinuria,  ± to 1+; severe proteinuria, 2+),^20^ platelet count, electrolyte levels, low‐density lipoprotein levels, and oral medications before surgery (angiotensin‐converting enzyme inhibitor or angiotensin receptor inhibitors [ACEI/ARB], statins, nonsteroidal anti‐inflammatory drugs). Intraoperative data included surgery type, aortic cross‐clamping time, CPB time, IABP use, and RBC transfusion. Postoperative data included rethoracotomy, oral ACEI/ARB, postoperative IABP, postoperative RBC transfusion, and prolonged mechanical ventilation (defined as the duration of mechanical ventilation more than 24 h).

### 2.3 Outcomes

The primary endpoint was AKI after cardiac surgery, which was defined based on the Kidney Disease Improving Global Outcomes (KDIGO) criteria,^21^ which is an elevation in serum creatinine of ≥0.3 mg/dl (≥26.5 µmol/L) within 48 h after surgery or an elevation in serum creatinine by 50% from baseline within 7 days after surgery. The last preoperative serum creatinine was used as the baseline.

### 2.4 Sample size

According to the rule of thumb that a minimum of five events is required for every predictor variable in a logistic model,^22^ we estimated that at least 545 patients were required in the development set for 60 candidate predictor variables, with an assumed event rate of 55%.

### 2.5 Statistical analysis

Data were collected using a standardized form for each operation in which the designated recorder was present. All data were entered in Epidata 3.1 (The EpiData Association, Odense, Denmark). Continuous variables are expressed as median (interquartile range) or mean ± standard deviation (*SD*), and Mann–Whitney *U* test or Student's *t*‐test was used for statistical comparisons between groups. Categorical variables are expressed as frequency (percentage), and Fisher exact test or χ^2^ test was used for statistical comparisons between groups. The continuous predictors (platelet count, albumin level, natremia, calcium level, magnesemia, and phosphorus level) were truncated at the 1st and 99th percentiles to limit the influence of extreme values. For missing data, multiple imputations with chain equations and an iteration of 10 times was used to estimate the missing data and were merged according to Rubin's rules.^23^ Then, multicollinearity between variables was tested with variance inflation factors. The Box–Tidwell method was used to test the linear correlations between continuous variables and the risk of AKI.

Least absolute shrinkage and selection operator (LASSO) regression was used to reduce the dimensions of the data and select variables in the development set. This approach avoids issues of multicollinearity and overfitting, even with a high number of potential predictors and a small sample size. Ten‐fold cross‐validation and the 1‐*SE* rule were performed to control for overfitting.^24^ The final variables were included in the logistic regression, and a new model was developed. To facilitate its clinical use, a nomogram was drawn based on the weight of each variable in the final multivariable regression model. The weighted point was calculated by the beta coefficient of each variable in the model. The variable with the highest beta coefficient was scored on a 100 points scale, and the remaining variables were scored according to their individual weighted effect. Finally, the total number of points was calculated.^25^

We validated the new model using the bootstrap method^26^ in the development set with an iteration of 1000 times. The performance of the new model, focusing on discrimination, calibration, and clinical usefulness, was also analyzed in the validation set. The performance of this new model to predict AKI was compared with that of the SRI score and Cleveland Clinic score in the validation set. The SRI score and Cleveland Clinic score were calculated based on the data included for the validation set. The definition of each variable used for scoring adopted the original standard^9^, ^11^ (Table S1). The area under the receiver operating characteristic curve (AUC) was used to assess discrimination. A calibration curve was plotted to evaluate the calibration and was accompanied by the Hosmer–Lemeshow test. Decision curve analysis (DCA) was used to evaluate the clinical usefulness of the model by quantifying the net benefits at different threshold probabilities in the validation set.^27^ The DeLong method was used to compare the AUC of each model.^28^

All analyses and reports for the development and validation of this model followed the Transparent Reporting of a multivariable prediction model for Individual Prognosis Or Diagnosis guidelines. All statistical analyses were carried out with IBM SPSS v.25.0 (SPSS IBM) and R software (version 3.6.1; https://www.r-project.org). A p < .05 was considered statistically significant.

## 3 RESULTS

### 3.1 Demographic and baseline clinical data of participants

The demographic and baseline clinical data of both the groups are presented in Table 1. Both the development set and the validation set had an incidence of AKI of 61.8%.

**Table 1:** Baseline characteristics of development and validation data set

Variables	Development set (*n* = 597)	Validation set (*n* = 251)	*p* value
Preoperative			
Age (years)	65 (62, 68)	64 (62, 68)	.723
Gender (male)	325 (54.4)	135 (53.8)	.861
Current smoking	47 (7.9)	12 (4.8)	.106
Height (cm)	160 (153, 167.5)	160 (156, 166.5)	.694
Weight (kg)	56 (50, 63)	56 (49, 65)	.828
Serum creatinine (µmol/L)	91 (78, 107.5)	90 (78, 105)	.483
eGFR (ml/min/1.73 m^2^)^a^	67.3 ± 16.2	68.0 ± 15.5	.564
eGFR ≤ 60 ml/min/1.73 m^2^	185 (31.0)	67 (26.7)	.212
Hypertension	181 (30.3)	74 (29.5)	.808
Diabetes mellitus	63 (10.6)	30 (12.0)	.552
COPD	15 (2.5)	11 (4.4)	.149
Cerebrovascular disease	47 (7.9)	27 (10.8)	.174
Peripheral vascular disease	9 (1.5)	4 (1.6)	.926
Previous cardiac surgery	48 (8.0)	11 (4.4)	.056
Recent myocardial infarction	14 (2.3)	10 (4.0)	.189
Contrast media exposure	240 (40.2)	115 (45.8)	.130
NYHA classification III or IV	329 (55.1)	155 (61.8)	.074
LVEF (%)	64 (54−70)	63 (53−68)	.215
Anemia	229 (38.4)	82 (32.7)	.117
Platelet (×10^9^/L)	190 (149, 229)	184 (151, 229)	.562
Total protein (g/L)^a^	67.2 ± 7.0	67.1 ± 7.7	.848
Albumin (g/L)^a^	36.3 ± 4.8	36.2 ± 5.5	.887
Total bilirubin (μmol/L)	16.6 (12.0, 22.2)	16.6 (12.6, 21.8)	.803
Indirect bilirubin (μmol/L)	4.6 (3.6, 6.0)	4.7 (3.7, 6.0)	.905
Aspartate aminotransferase (U/L)	26 (22, 34)	26 (22, 34)	.532
Alanine aminotransferase (U/L)	21 (16, 29)	20 (15, 29)	.402
Alkaline phosphatase (U/L)	61 (50, 77)	60 (50, 71)	.274
Natremia (mmol/L)	138.6 (136.3, 140.3)	138.5 (136.8, 140.5)	.690
Potassium (mmol/L)	3.9 (3.7, 4.2)	3.9 (3.7, 4.2)	.451
Magnesemia (mmol/L)	0.9 (0.8, 0.9)	0.9 (0.8, 0.9)	.220
Calcium (mmol/L)	2.3 (2.2, 2.3)	2.2 (2.2, 2.3)	.236
Phosphorus (mmol/L)	1.2 (1.1, 1.3)	1.2 (1.1, 1.4)	.241
Glucose (mmol/L)	5.2(4.8, 6.0)	5.2 (4.7, 6.0)	.579
Total cholesterol (mmol/L)	4.6 (3.9, 5.4)	4.6 (4.0, 5.4)	.587
Triglyceride (mmol/L)	1.1 (0.8, 1.5)	1.1 (0.8, 1.7)	.069
High‐density lipoprotein (mmol/L)	1.1 (0.9, 1.4)	1.1 (0.9, 1.4)	.972
Low‐density lipoprotein (mmol/L)	2.7 (2.1, 3.4)	2.7 (2.2, 3.3)	.659
CO_2_CP (mmol/L)	26.8 (25.0, 28.5)	26.5 (24.7, 28.0)	.093
Proteinuria			.098
No proteinuria	504 (88.4)	209 (83.3)	
Mild proteinuria	77 (12.9)	28 (11.2)	
Severe proteinuria	16 (2.7)	14 (5.6)	
Uric acid (μmol/L)	407.5 (341.0, 488.5)	418 (346, 481)	.441
International normalized ratio	1.1 (1.0, 1.2)	1.1 (1.0, 1.2)	.228
IABP	17 (2.8)	6 (2.4)	.708
Emergency	21 (3.5)	14 (5.6)	.169
Preoperative drugs use			
Antibiotic	89 (14.9)	41 (16.3)	.599
ACEI/ARB	263 (44.1)	106 (42.2)	.625
Vasoactive drug	12 (2.0)	4 (1.6)	.684
Statins	117 (19.6)	60 (23.9)	.159
NSAID	42 (7.0)	17 (6.8)	.891
Intraoperative variables			
Procedure			.762
CABG	29 (4.9)	14 (5.6)	
Valve	447 (74.9)	182 (72.5)	
Valve and CABG	121 (20.3)	55 (21.9)	
CPB time > 120 min	287 (48.1)	114 (45.4)	.480
Aortic cross‐clump time > 80 min	261 (43.7)	108 (43.0)	.853
Erythrocyte transfusion (U)	2 (1, 3)	2 (1, 3)	.581
IABP	35 (5.9)	13 (5.2)	.694
Postoperative variables			
Resurgery	40 (6.7)	21 (8.4)	.391
Erythrocyte transfusion (U)	0.5 (0, 2)	0 (0, 2)	.875
Postoperative drugs use			
Antibiotic	593 (99.3)	251 (100)	.194
ACEI/ARB	77 (12.9)	31 (12.4)	.827
Vasoactive drug	345 (57.8)	135 (53.8)	.283
IABP	42 (7.0)	16 (6.4)	.728
Prolonged MV	207 (34.7)	85 (33.9)	.821
CVP > 10 cmH_2_O	381 (63.8)	164 (65.3)	.673
AKI	369 (61.8)	155 (61.8)	.988
Inhospital mortality	19 (3.2)	7 (2.8)	.761

Abbreviations: ACEI/ARB, angiotensin‐converting anzyme inhibitior/angiotensin receptor blocker; AKI, acute kidney injury; CABG, coronary artery bypass grafting; CO_2_CP, carbon dioxide combining power; COPD, chronic obstructive pulmonary disease; CPB, cardiopulmonary bypass; CVP, central venous pressure; eGFR, estimated glomerular filtration rate; IABP, intra‐aortic balloon pump; LVEF, left ventricular ejection fraction; MV, mechanical ventilation; NSAID, nonsteroidal anti‐inflammatory drugs; NYHA, New York Heart Association.

^a^ Values are expressed in mean ± standard deviation.

### 3.2 Feature selection and model construction

**Figure 2:**
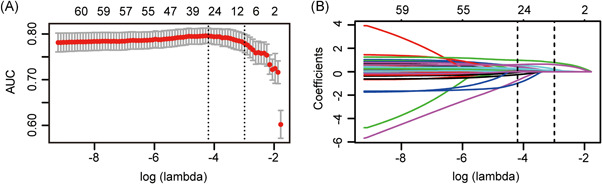
Predictor selection using the LASSO regression method. (A) Ten‐fold cross‐validation via minimum criteria was used to select the Tuning parameter (λ) in the LASSO model. The AUC was plotted versus log (λ). The dotted vertical lines were plotted at the optimal values using the minimum criteria and the one standard error of the minimum criteria (the 1−*SE* criteria). The log (λ) of −2.987 and the λ value of 0.0505 were selected. (B) A coefficient profile plot was produced against the log (λ) sequence. The dotted vertical lines were plotted at the optimal values using the 1−*SE* criteria. Predictors were selected based on the 1−*SE* criteria, where optimal λ resulted in seven nonzero coefficients. AUC, area under the receiver operating characteristic curve; LASSO, Least absolute shrinkage and selection operator

**Figure 3:**
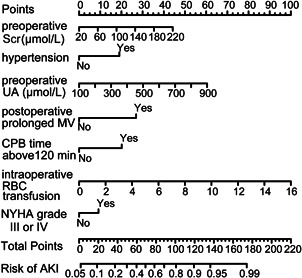
Nomogram for predicting AKI after cardiac surgery in patients of advanced age. AKI, acute kidney injury; CPB, cardiopulmonary bypass; MV, mechanical ventilation; RBC, red blood cells; Scr, serum creatinine; UA, uric acid

Several variables were correlated with a higher risk of AKI according to the univariate analysis in the development set (Table S2). To generate this predictive model for the risk of AKI, we incorporated a total of 60 potential variables in LASSO regression analysis and selected seven best predictors such as preoperative baseline serum creatinine, hypertension, preoperative uric acid level, NYHA class III and above, CPB time > 120 min, intraoperative RBC transfusion, and postoperative prolonged mechanical ventilation (Figure 2). These variables were all included to develop a model for predicting the risk of AKI through logistic regression. The detailed parameters of the variables in the model are presented in Table 2. A nomogram was also drawn according to the logistic regression results (Figure 3).

**Table 2:** Multivariate logistic regression analysis of variables selected with LASSO for predicting AKI

Variables	*β*	*SE*	*P*	OR	95% CI
Preoperative					
Serum creatinine (µmol/L)	0.010	0.005	.051	1.010	1.000, 1.020
Hypertension	0.831	0.224	<.001	2.296	1.479, 3.564
Uric acid (μmol/L)	0.003	0.001	.001	1.003	1.001, 1.005
NYHA classification III or IV	0.400	0.198	.044	1.492	1.011, 2.200
Intraoperative					
CPB time >120 min	0.881	0.210	<.001	2.412	1.599, 3.639
Erythrocyte transfusion (U)	0.273	0.074	<.001	1.314	1.137, 1.518
Postoperative					
Prolonged MV	1.178	0.245	<.001	3.247	2.008, 5.249
Constant	−3.538	0.559	<.001	0.029	

Abbreviations: CI, confidence interval; CPB, cardiopulmonary bypass; cardiopulmonary bypass; MV, mechanical ventilation; NYHA, New York Heart Association; OR, odds ratio; *SE*, standard error.

### 3.3 Model validation and comparison

**Figure 4:**
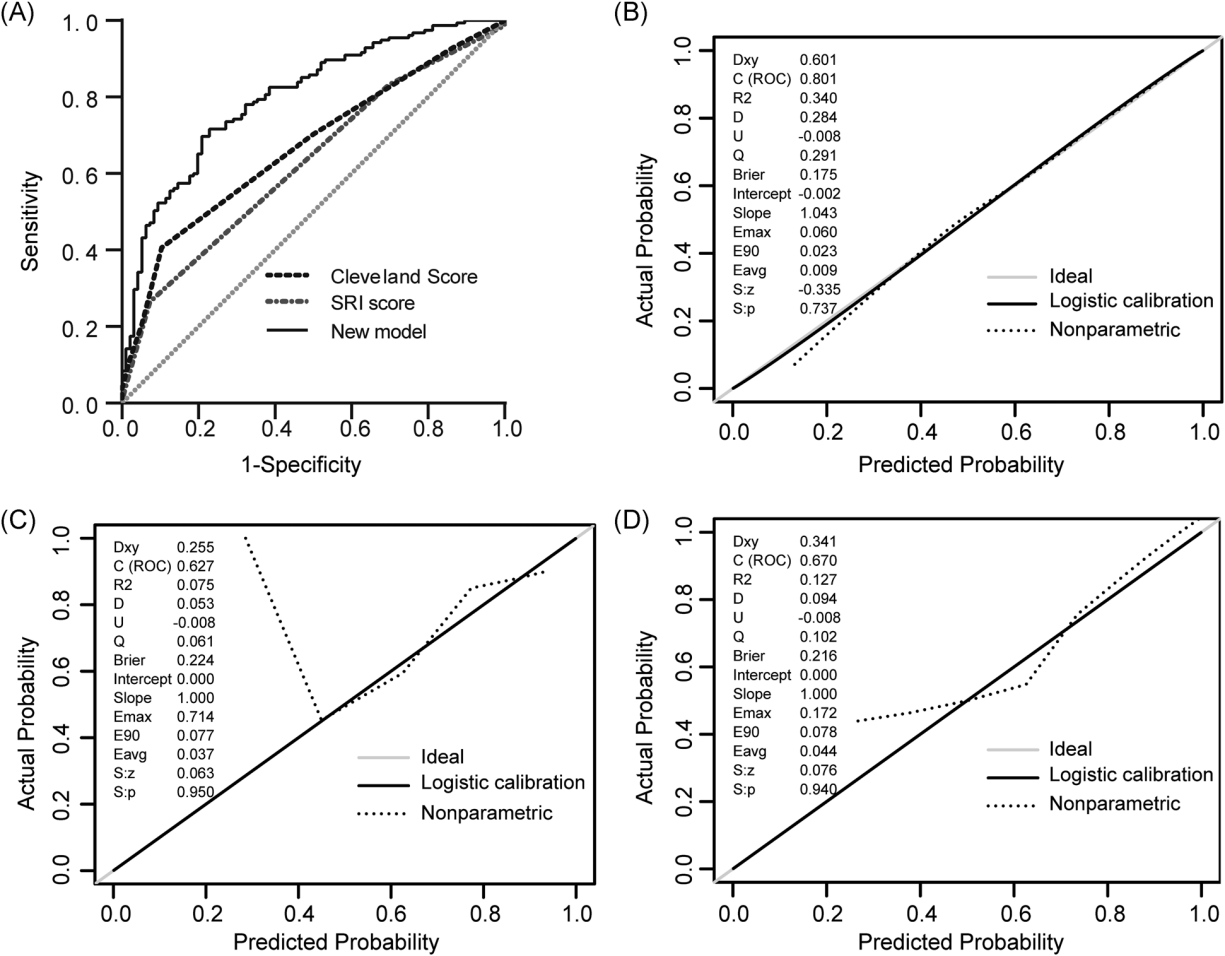
The AUC and calibration curves for models in the validation set. (A) Comparison of AUC among models for postoperative AKI. New model AUC: 0.801 (0.746–0.856); Cleveland Clinic score AUC: 0.670 (0.604–0.737); SRI score AUC 0.627 (0.558–0.697); the new model versus Cleveland score, *p* = .001; new model versus SRI score, *p* < .001. (B–D) Calibration curves for the new model, SRI score, and Cleveland score, respectively. Calibration plots illustrate the relationship between the predicted AKI risk according to the models and the actual occurrence of AKI in the validation data. A plot along the 45‐degree line represents a calibration of the model in which the predicted probabilities are identical to the actual outcomes. The dotted line has a close fit to the solid line, which indicated better predictive accuracy of the model. AKI, acute kidney injury; AUC, area under the receiver operating characteristic curve; SRI, Simplified Renal Index

**Figure 5:**
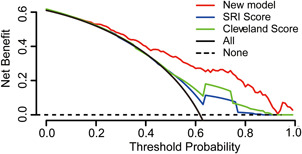
Decision curve analyses for prediction models. The *x*‐axis shows the threshold probability. The *y*‐axis shows the net benefit. The dashed and solid black lines represent the hypothesis that no patients and all patients had AKI, respectively. The net benefit was computed by subtracting the proportion of false positives from the proportion of true positives in all patients, with weighting the relative harm driven by the false positive. The threshold probability was where the expected benefit of avoiding treatment is equal to the expected benefit of treatment. For each decision threshold, the net benefits of the new model, Cleveland score, and SRI score are presented. For a given threshold, the difference in net benefit between two scores was the additional number of AKI cases identified (per 1000) without increasing the number of false‐positive classifications. Across the range of decision thresholds, the new model was consistently positive and had a larger net benefit than the SRI and Cleveland scores. AKI, acute kidney injury; SRI, Simplified Renal Index

We initially validated the new model in the development set. The model demonstrated good discrimination with an AUC of 0.804 (95% confidence interval [CI], 0.769–0.840). After correction by the bootstrap method, the AUC was 0.797 (95% CI, 0.762–0.835). We also compared the performance of our new model with that of the SRI score and Cleveland Clinic score in the validation set. The AUC and calibration curves for models are shown in Figure 4. The AUC for the new model, Cleveland Clinic score, and SRI score were 0.801 (95% CI, 0.746–0.856), 0.670 (95% CI, 0.604–0.737), and 0.627 (95% CI, 0.558–0.697), respectively (A). Although the Hosmer–Lemeshow test demonstrated nonsignificant statistical value for each model (0.835, 0.977, and 0.725, respectively), the calibration curves showed that the calibration of the new model was better than those of the SRI score and Cleveland Clinic score (B,D). The DCA curve showed that within the entire range of prediction thresholds, using the new model to predict AKI risk obtained net benefits, and in most of the threshold ranges, this model obtained greater clinical net benefits in comparison with Cleveland Clinic score or SRI score (Figure 5).

## 4 DISCUSSION

In this study, we developed a new model for predicting AKI after cardiac surgery in patients of advanced age. Compared with the SRI score and Cleveland Clinic score, in terms of the discrimination, calibration, and clinical usefulness, our new model may be more suitable for predicting AKI among patients of advanced age undergoing cardiac surgery.

The previous models^8^, ^9^, ^10^, ^11^, ^12^ for predicting AKI were generally evaluated in terms of discrimination and calibration. Whether the decisions made based on these models could generate clinical benefits were not assessed. The DCA curve is a novel method for evaluating the clinical utility of a model by quantifying the net benefits, which may aid clinical decision‐making.^27^ We found that our new model exhibited better performance than the Cleveland Clinic score or SRI score. The reason for the improved performance of our new model is likely the inclusion of new important risk factors, including the preoperative uric acid level and intraoperative RBC transfusion, which were not considered in these models.

Recent studies have suggested that preoperative uric acid elevation is an independent risk factor for AKI in patients undergoing cardiac surgery.^14^ Consistently, we also observed this. Uric acid causes kidney damage through multiple mechanisms, including inhibiting the proliferation and migration of endothelial cells, promoting the apoptosis of proximal renal tubules and vascular endothelial cells, activating the renin‐angiotensin system to induce vasoconstriction, increasing reactive oxygen radical levels, and promoting the release of inflammatory mediators.^29^ Some studies have shown that with modern economic development and lifestyle changes, the prevalence of hyperuricemia in developing countries has increased, especially in an advanced age.^30^ Therefore, we believed it was imperative to include the serum uric acid level as a variable for generating our model.

Although RBC transfusion can increase tissue oxygen supply and subsequently improve organ function, some studies have reported that RBC transfusion is also a risk factor for AKI in patients after cardiac surgery.^15^ A meta‐analysis indicated that for each additional unit of perioperative RBCs transfused, the AKI risk after cardiac surgery is increased by 10%–20%.^31^ Moreover, older patients are more likely to need RBC transfusion during heart surgery than younger patients,^32^ and thus, may have a higher risk of developing AKI. The exact mechanisms whereby RBC transfusion causes renal damage are not clear, but several explanations have been proposed. For example, stored RBCs may have an impaired capability of oxygen delivery and predisposition to oxidative stress.^31^ Therefore, we believe that including RBC transfusion as a variable in our model development is reasonable.

The predictors in our model, such as baseline serum creatinine, NYHA class III or IV, CPB time above 120 min, and hypertension, have been identified as risk factors for AKI after cardiac surgery in previous studies.^8^, ^10^, ^11^, ^17^ Mechanical ventilation duration was another AKI risk factor. The pathogenic mechanism for mechanical ventilation may involve the reduction of cardiac output, induction of the release of inflammatory factors, and redistribution of renal blood flow, thus causing the organ's injury.^33^ A duration of mechanical ventilation longer than 24 h increased the risk of AKI by three‐fold.^34^ In our study, prolonged mechanical ventilation (defined as mechanical ventilation for more than 24 h) was predictive of AKI.

The incidence of AKI in this study was 61.8%, which was higher than that reported in European and American populations.^35^, ^36^ The following explanations may account for this discrepancy: (1) in our study, over 70% of cardiac surgeries were valve surgeries, while most of the European and American patients received CABG surgery. Compared with CABG surgery, the pathophysiology of valve surgery is more complex and diverse. Patients with valvular disease often have low cardiac output and are more prone to AKI during cardiac surgery; (2) the medical resources in China are not evenly distributed, and the rate of early diagnosis of heart diseases is lower than that in the Europe and United States; and (3) patients of advanced age often have multiple comorbidities, such as hypertension and cardiac insufficiency, which are usually accompanied by damage of kidney function. The incidence revealed in this study was consistent with a previous report, which found that the AKI incidence in adult Chinese patients undergoing cardiac surgery was higher than that in European and American patients.^37^

Some limitations of this study should be noted. First, although the Department of Cardiac Surgery in our hospital is the largest heart surgery center in Southern China, the data used to develop this new model still originated from a single center. Due to the different characteristics of the population, whether our conclusions can be extrapolated to other populations must be validated in the future. Second, this was a retrospective study and exhibited some unavoidable bias associated with the nature of this study. For example, the information regarding the duration of IABP, presence of coronary artery lesions, amount of bleeding, and partial medication use, such as inotropic agents, was not available and thus was not included in our study. Lastly, we only used the serum creatinine recommended by KDIGO, but not urine volume, as the diagnostic criterion for AKI. Because urine volume is easily affected by a number of factors, such as diuretic use, fluid replacement, and urine collection, most studies on AKI only use serum creatinine as a diagnostic criterion.^36^

## 5 CONCLUSION

We developed a model for predicting AKI after cardiac surgery in patients of advanced age. Compared with the SRI score and Cleveland Clinic score, this new model exhibited better performance. In the future, we will integrate this model into our electronic medical records system to facilitate its clinical application.

## CONFLICT OF INTERESTS

The authors declare that there are no conflict of interests.

## AUTHOR CONTRIBUTIONS

Penghua Hu, Yuanhan Chen, and Xinling Liang were responsible for the conception and design of the study. Penghua Hu, Yanhua Wu, Li Song, Li Zhang, Zhilian Li, and Lei Fu were responsible for the acquisition and analysis of data; furthermore, Penghua Hu, Yuanhan Chen, and Shuangxin Liu were in charge of statistical analysis. Penghua Hu and Yuanhan Chen drafted the manuscript; Zhiming Ye, Wei Shi, and Xinling Liang revised and commented the draft. Xinling Liang supervised the entire process, gave constructive advice, and acquired funding. All authors read and approved the final version of the manuscript.

## Supporting information

Supplementary information.

Supplementary information.

Supplementary information.

## Data Availability

The data that support the findings of this study are available from the corresponding author upon reasonable request.
