# Supplementary material for: Development and validation of a model for predicting acute kidney injury after cardiac surgery in patients of advanced age
Source: J Card Surg. 2020 Dec 12;36(3):806–14. doi: 10.1111/jocs.15249 (PMC7898501; doi:10.1111/jocs.15249)
Supplement: Supplementary file 1 — Supplementary information. [file JOCS-36-806-s001.docx]

**SUPPLEMENTARY MATERIAL**

**TABLE S1** Scoring models and definition of variables

|  | Cleveland Score^a^ | |  | SRI Score^b^ | |
| --- | --- | --- | --- | --- | --- |
|  | Definition | Points |  | Definition | Points |
| Variables |  |  |  |  |  |
| Gender | Female | 1 |  |  |  |
| Chronic heart failure | Yes | 1 |  |  |  |
| COPD | Yes | 1 |  |  |  |
| LVEF | <35% | 1 |  | ≦40% | 1 |
| Preoperative IABP | Yes | 2 |  | Yes | 1 |
| Diabetes mellitus | Insulin requiring | 1 |  | Requiring medication | 1 |
| Preoperative kidney function | serum creatinine<1.2 mg/dL | 0 |  | eGFR^c^ >60 ml/min | 0 |
|  | serum creatinine, 1.2-2.1 mg/dL | 2 |  | eGFR, 31-60 ml/min | 1 |
|  | serum creatinine ≧2.1 mg/dL | 5 |  | eGFR≦30 ml/min | 2 |
| Previous surgery | Yes | 1 |  | Yes | 1 |
| Timing of surgery | Emergency | 2 |  | Nonelective | 1 |
| Type of surgery | CABG only | 0 |  | Isolated CABG or ASD | 0 |
|  | Valve only | 1 |  | Other than isolated CABG or ASD | 1 |
|  | Combined (valve plus CABG) or other | 2 |  |  |  |
| Score range |  | 0-17 |  |  | 0-8 |

Abbreviations: ASD, atrial septal defect; CABG, coronary artery bypass graft; COPD, chronic obstructive pulmonary disease; eGFR, estimated glomerular filtration rate; IABP, intra-aortic balloon pump; LVEF, left ventricular ejection fraction.

^a^ Model provided by Thakar et al. includes 10 variables and 13 definitions.

^b^Model provided by Wijeysundera et al. includes 7 variables and 8 definitions.

^c^eGFR were derived from Cockcroft and Gaul.

**TABLE S2** Univariate analysis between candidate variables and AKI in development group after multiple imputation

| Variables | Non-AKI (n=228) | AKI (n=369) | *P* value |
| --- | --- | --- | --- |
| Preoperative |  |  |  |
| Gender (male) | 100 (43.9) | 225 (61.0) | <0.001 |
| Age (years) | 64.0 (62.0, 67.0) | 65.0 (62.0, 68.0) | 0.012 |
| BMI group (Kg/m^2^) |  |  | 0.622 |
| <18.5 | 36 (15.8) | 51 (13.8) |  |
| 18.5—30 | 189 (82.9) | 310 (84.0) |  |
| ≥30 | 3 (1.3) | 8 (2.2) |  |
| Serum creatinine (µmol/L) | 84.5 (73.0, 96.7) | 96.0 (83.0, 111.0) | <0.001 |
| eGFR ≤ 60ml/min/1.73m^2^ | 46 (20.2) | 139 (37.7) | <0.001 |
| Current smoking | 11 (4.8) | 36 (9.8) | 0.030 |
| Hypertension | 47 (20.6) | 134 (36.3) | <0.001 |
| Diabetes mellitus | 17 (7.5) | 46 (12.5) | 0.053 |
| COPD | 3 (1.3) | 12 (3.3) | 0.142 |
| Cerebrovascular disease | 11 (4.8) | 36 (9.8) | 0.030 |
| Peripheral vascular disease | 3 (1.3) | 6 (1.6) | 0.762 |
| Previous cardiac surgery | 10 (4.4) | 38 (10.3) | 0.010 |
| Recent myocardial infarction | 2 (0.9) | 12 (3.3) | 0.062 |
| Contrast media exposure | 79 (34.6) | 161 (43.6) | 0.030 |
| NYHA classification III or IV | 102 (44.7) | 227 (61.5) | <0.001 |
| LVEF ≤ 40% | 10 (4.4) | 32 (8.7) | 0.047 |
| Anemia | 73 (32.0) | 156 (42.3) | 0.012 |
| Platelet (×10^9^/L) | 191.5 (148.5, 230.0) | 189.0 (152.0, 226.0) | 0.865 |
| Total protein (g/L) | 68.0 (7.0) | 66.8 (7.0) | 0.047 |
| Albumin (g/L) | 37.2 (4.6) | 35.7 (4.7) | <0.001 |
| Total bilirubin (μmol/L) | 17.0 (12.2, 22.8) | 16.5 (12.0, 21.4) | 0.360 |
| Indirect bilirubin (μmol/L) | 4.6 (3.6, 6.0) | 4.6 (3.6, 6.0) | 0.942 |
| Aspartate aminotransferase (U/L) | 27.0 (22.0, 33.0) | 27.0 (22.0, 33.0) | 0.820 |
| Alanine aminotransferase (U/L) | 21.0 (16.0, 27.0) | 21.0 (16.0, 30.0) | 0.774 |
| Alkaline phosphatase (U/L) | 64.5 (54.5, 78.0) | 62.0 (50.0, 73.0) | 0.071 |
| Natremia (mmol/L) | 138.6 (136.6, 140.4) | 138.5 (136.1, 140.2) | 0.291 |
| Potassium (mmol/L) | 3.9 (3.6, 4.2) | 3.9 (3.7, 4.2) | 0.083 |
| Magnesemia (mmol/L) | 0.9 (0.8, 0.9) | 0.9 (0.8, 0.9) | 0.277 |
| Calcium (mmol/L) | 2.3 (2.2, 2.3) | 2.2 (2.2, 2.3) | 0.046 |
| Phosphorus (mmol/L) | 1.2 (1.1, 1.3) | 1.2 (1.1, 1.3) | 0.738 |
| Glucose (mmol/L) | 5.2 (4.8, 5.9) | 5.2 (4.8, 6.1) | 0.527 |
| Total cholesterol (mmol/L) | 4.7 (4.2, 5.2) | 4.7 (4.0, 5.1) | 0.443 |
| Triglyceride (mmol/L) | 1.3 (0.9, 1.4) | 1.2 (0.9, 1.3) | 0.395 |
| High density lipoprotein (mmol/L) | 1.2 (1.0, 1.3) | 1.2 (0.9, 1.3) | 0.401 |
| Low density lipoprotein (mmol/L) | 2.7 (2.4, 3.2) | 2.7 (2.2, 3.1) | 0.325 |
| CO_2_CP (mmol/L) | 27.0 (25.1, 28.7) | 26.7 (24.8, 28.3) | 0.052 |
| Uric acid (μmol/L) | 388.0 (310.0, 448.5) | 425.0 (355.0, 513.0) | <0.001 |
| International Normalized Ratio | 1.1 (1.0, 1.2) | 1.1 (1.0, 1.2) | 0.143 |
| Proteinuria |  |  | <0.001 |
| no proteinuria | 209 (91.7) | 295 (79.9) |  |
| mild proteinuria | 17 (7.5) | 60 (16.3) |  |
| severe proteinuria | 2 (0.9) | 14 (3.8) |  |
| Erythrocyte transfusion (U) | 0.0 (0.0, 0.0) | 0.0 (0.0, 0.0) | 0.733 |
| IABP | 2 (0.9) | 15 (4.1) | 0.023 |
| Emergency | 5 (2.2) | 16 (4.3) | 0.167 |
| Preoperative drugs use |  |  |  |
| antibiotic | 34 (14.9) | 55 (14.9) | 0.998 |
| ACEI/ARB | 83 (36.4) | 180 (48.8) | 0.003 |
| Statins | 31 (13.6) | 86 (23.3) | 0.004 |
| NSAID | 10 (4.4) | 32 (8.7) | 0.047 |
| Vasoactive drug | 6 (2.6) | 6 (1.6) | 0.395 |
| Intraoperative |  |  |  |
| Valve + CABG | 26 (11.4) | 95 (25.7) | <0.001 |
| CPB time > 120min | 61 (26.8) | 226 (61.2) | <0.001 |
| Aortic cross-clump time >80 min | 60 (26.3) | 201 (54.5) | <0.001 |
| IABP | 5 (2.2) | 30 (8.1) | 0.003 |
| Erythrocyte transfusion (U) | 2.0 (1.0, 2.0) | 2.0 (2.0, 4.0) | <0.001 |
| Postoperative |  |  |  |
| Resurgery | 11 (4.8) | 29 (7.9) | 0.150 |
| Postoperative drugs use |  |  |  |
| Antibiotic | 228 (100.0) | 365 (98.9) | 0.115 |
| ACEI/ARB | 43 (18.9) | 34 (9.2) | 0.001 |
| Vasoactive drug | 124 (54.4) | 221 (59.9) | 0.186 |
| IABP | 5 (2.2) | 37 (10.0) | <0.001 |
| Prolonged MV | 31 (13.6) | 176 (47.7) | <0.001 |
| CVP ≥10 cmH_2_O | 131 (57.5) | 250 (67.8) | 0.011 |
| Erythrocyte transfusion (U) | 0.0 (0.0, 1.0) | 1.0 (0.0, 2.0) | <0.001 |

Abbreviations: ACEI/ARB, angiotensin converting anzyme inhibitior / angiotensin receptor blocker; BMI, Body Mass Index; CABG, coronary artery bypass grafting; CO2CP, carbondioxide combining power; COPD, chronic obstructive pulmonary disease; CPB, cardiopulmonary bypass; CVP, central venous pressure; eGFR, estimated glomerular filtration rate; IABP, intra-aortic balloon pump; LVEF, left ventricular ejection fraction; MV, mechanical ventilation; NSAID, non-steroidal anti-inflammatory drugs; NYHA, New York Heart Association.
