# Supplementary material for: Development and validation of a model for predicting acute kidney injury after cardiac surgery in patients of advanced age
Source: J Card Surg. 2020 Dec 12;36(3):806–14. doi: 10.1111/jocs.15249 (PMC7898501; doi:10.1111/jocs.15249)
Supplement: Supplementary file 2 — Supplementary information. [file JOCS-36-806-s002.pdf]

Certification

Manuscript ID: JOCS-2020-OA-1367

Journal of Cardiac Surgery

Dear editor:

The Manuscript ID: JOCS-2020-OA-1367, **Titled** "Development and validation of a model for predicting acute kidney injury after cardiac surgery in patients of advanced age" **has been submitted to the professional statistical department for analysis and review. The statistical analysis method is reliable and the processing of the data are detailed and reliable.**

Sincerely,

Nutritional Epidemiology Institute, Tianjin Medical University, Tianjin, China.

天津医科大学

营养流行病学研究所
